# Gravity complexes as a focus of seafloor fluid seepage: the Rio Grande Cone, SE Brazil

**DOI:** 10.1038/s41598-023-31815-1

**Published:** 2023-03-21

**Authors:** M. Ketzer, D. Praeg, A. H. Augustin, L. F. Rodrigues, A. K. Steiger, M. Rahmati-Abkenar, A. R. Viana, D. J. Miller, A. Malinverno, G. R. Dickens, J. A. Cupertino

**Affiliations:** 1 Department of Biology and Environmental Science, Linnaeus University, 391 81 Kalmar, Sweden; 2 Géoazur, 250 Rue Albert Einstein, 06560 Valbonne, France; 3 Pontificia Universidade Catolica do Rio Grande do Sul, Porto Alegre, 91619-900 Brazil; 4 Universidade Federal do Rio Grande, Rio Grande, 96203-900 Brazil; 5 Petrobras Petroleo Brasileiro SA, Rio de Janeiro, 20031-170 Brazil; 6 Lamont-Doherty Earth Observatory of Columbia University, Palisades, NY 10964 USA; 7 Trinity College Dublin, Dublin 2, Ireland

**Keywords:** Solid Earth sciences, Geochemistry, Geology, Sedimentology

## Abstract

Seafloor methane emissions can affect Earth’s climate and ocean chemistry. Vast quantities of methane formed by microbial decomposition of organic matter are locked within gas hydrate and free gas on continental slopes, particularly in large areas with high sediment accumulations such as deep-sea fans. The release of methane in slope environments has frequently been associated with dissociation of gas hydrates near the edge of the gas hydrate stability zone on the upper slope, with discharges in greater water depths less understood. Here we show, using data from the Rio Grande Cone (western South Atlantic), that the intrinsic, gravity-induced downslope collapse of thick slope sediment accumulations creates structures that serve as pathways for gas migration, unlocking methane and causing seafloor emissions via giant gas flares in the water column. The observed emissions in the study region (up to 310 Mg year^−1^) are three times greater than estimates for the entire US North Atlantic margin and reveal the importance of collapsing sediment accumulations for ocean carbon cycling. Similar outgassing systems on the Amazon and Niger fans suggest that gravity tectonics on passive margins is a common yet overlooked mechanism driving massive seafloor methane emissions in sediment-laden continental slopes.

## Introduction

Seafloor methane emissions in deep-water environments (typically continental slopes and deeper, i.e., > 200 m) remain poorly understood and lack accurate estimates^1,2^. They probably represent a minor contribution to the global carbon cycle, especially in terms of direct atmospheric input^3,4^. However, seafloor methane emissions increase with escalating deep-sea temperatures, notably at the feather edge of the gas hydrate stability zone (GHSZ), potentially intensifing ocean acidification^5,6^. Prominent rapid negative carbon isotope excursions marking climate extremes in the geological record suggest massive methane release from deep-water systems in the past^7^.

Methane in deep-sea sediment mostly originates from microbial methanogenesis, a terminal biodegradation process that converts a fraction (e.g., 20%) of labile particulate organic carbon into gas^8^. This process is particularly important where sediment accumulates at high rates on continental slopes over long time scales, such as offshore the Amazon, Mississippi, and Nile rivers, which efficiently sequester large quantities of organic carbon^9,10^. The organic carbon stored in the entire Amazon deep-sea fan, for instance, exceeds the total quantity held by the present-day Amazon forest by several orders of magnitude^11^.

Methane formed in deep-water sediments can cycle between dissolved gas, free gas bubbles and solid gas hydrates, the latter occurring because relatively cold temperatures and high pressures between the seafloor and at the base of the gas hydrate stability zone (GHSZ)^12^. These solids host an enormous though poorly constrained mass of carbon in marine sediments worldwide (e.g., 550–56,000 GtC)^13–16^, and restrict methane fluxes from reaching the water column^17^. However, seafloor methane seeps are increasingly recognised as the termini of systems where structures create permeable pathways allowing fluid and gas to migrate across and from the GHSZ^18–21^. These seeps fuel benthic chemosynthetic communities that use methane and its oxidation by-products as source of energy^22^, and constitute the main conduit for transporting methane from underlying hydrocarbon accumulations to the deep ocean^19^.

Tilting and faulting of sedimentary units provide pathways for gas-rich fluids to migrate across the GHSZ^23^. Recognised mechanisms for faulting include salt diapirism^24^ and compressional tectonics^25,26^. In this paper, we reveal another mechanism, one operating in large, rapidly-deposited deep-sea sediment accumulations on passive margins. Based on geophysical and geochemical data from Rio Grande Cone (RGC), we show that gravity-induced downslope collapse of sediment accumulations, an intrinsic process involving thin-skinned upslope extension and downslope compression above one or more detachment surfaces^27^, produces faults that allow massive migration of gas-rich fluids across the GHSZ and the seafloor. Our findings invite further investigations of similar possible outgassing systems in dozens of other large, collapsing deep-sea fans around the world ^28^, and a possible revision of seafloor methane emissions estimates for deep-water settings^29^.

## Methane migration pathways in a collapsing depocenter

**Figure 1:**
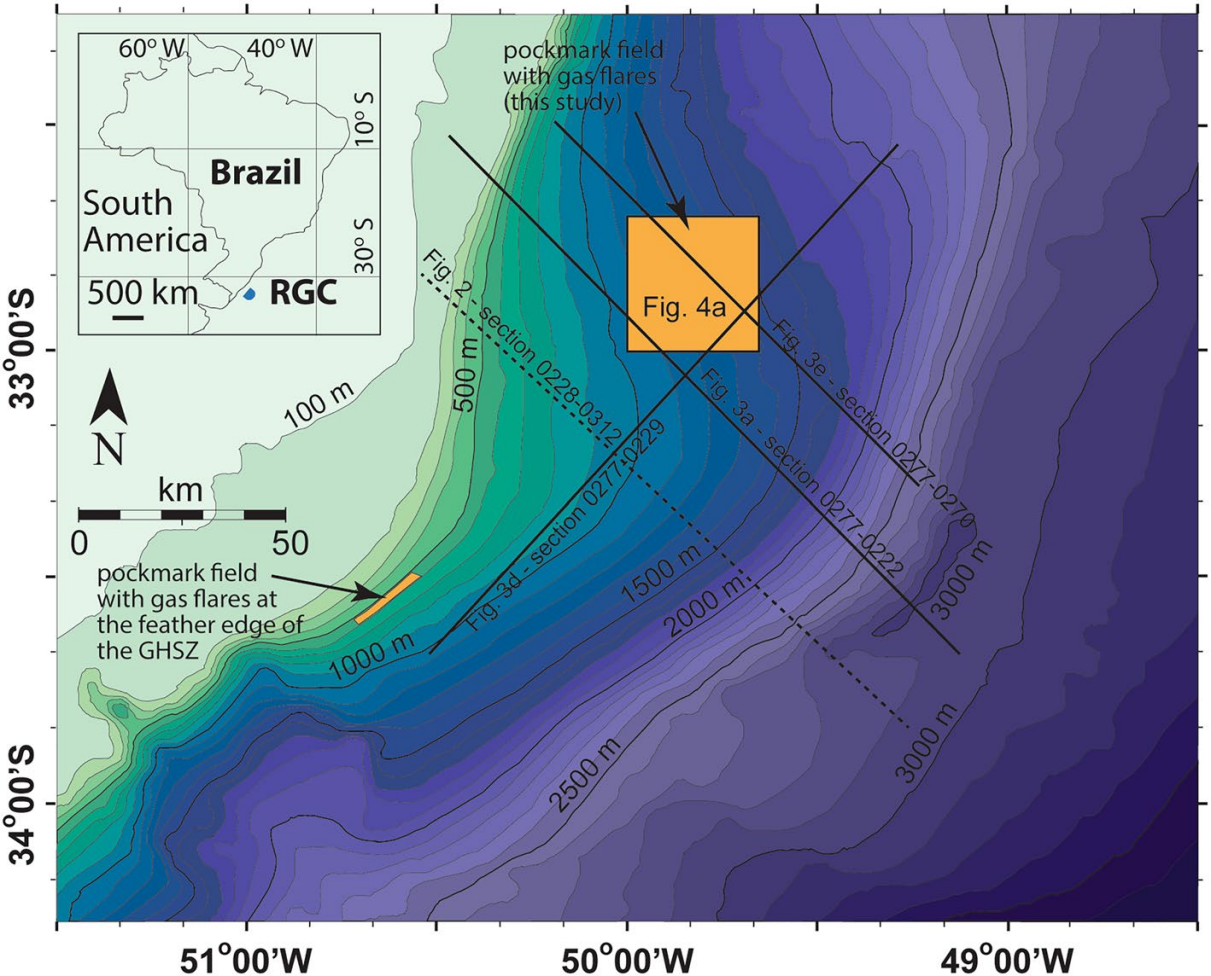
Bathymetric map obtained from a previous study in the area^37^ showing the location the Rio Grande Cone (RGC; inset) and seismic profiles used in this study (solid lines). The dashed line shows the location of the interpreted section in Fig. 2 showing the main structural framework of the RGC. Note the areas with the two pockmark fields previously recognized in the RGC^38^ (orange polygons). The depth range of the feather edge of the gas hydrate stability zone (GHSZ) is 550–585 m^37^.

**Figure 2:**
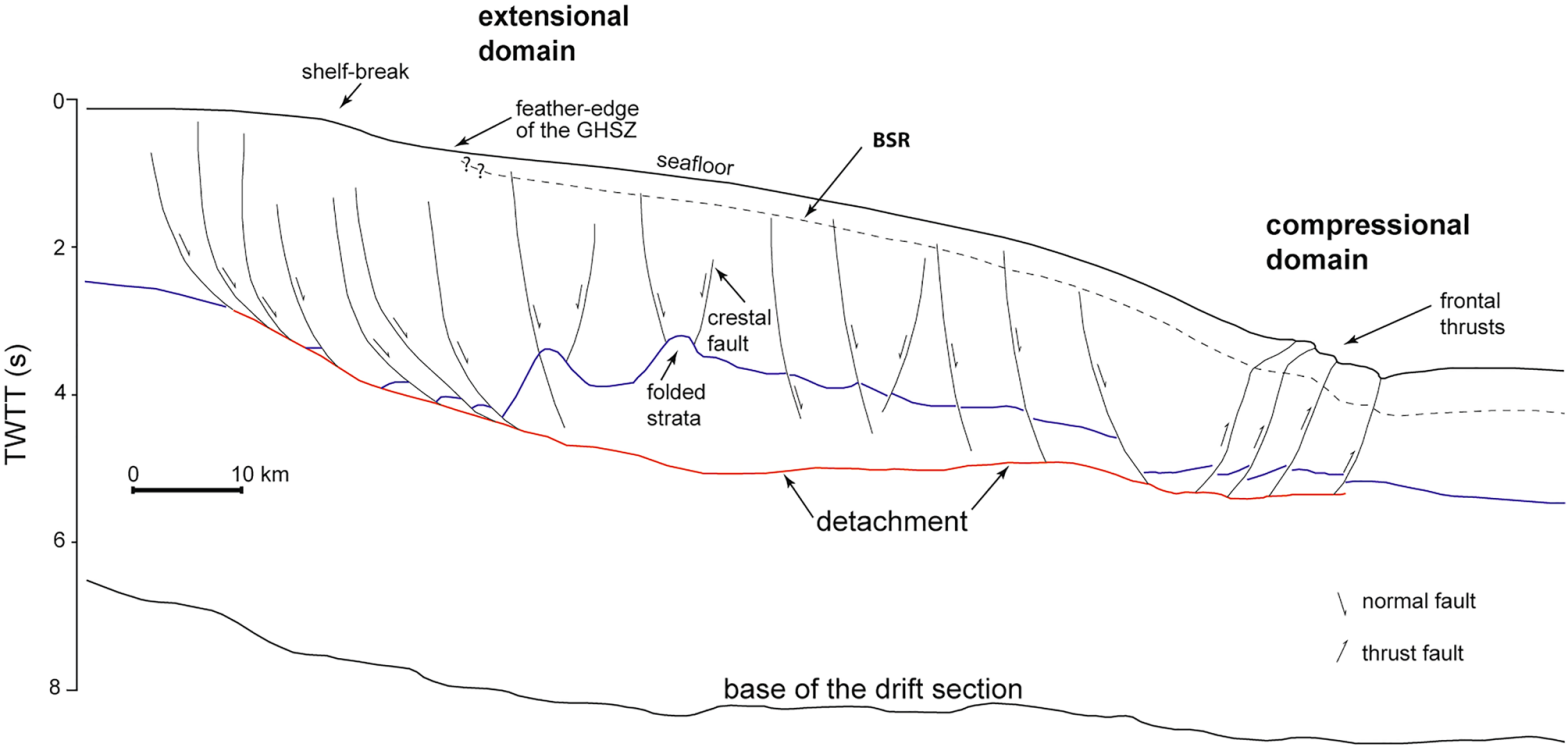
Line drawing showing the main structural framework of the Rio Grande Cone (RGC), the bottom simulating reflector (BSR) and the location of the feather-edge of the gas hydrate stability zone (GHSZ). The base of the drift section of the South Atlantic in the RGC is Albian/Cenomanian in age^39^, while the detachment (in red) cuts Lower Miocene shales at the centre of the figure (ca. 5 s). The blue line represents the top of Middle-Upper Miocene deposits. Line drawings were obtained from an interpreted seismic profile of a previous work in the area^40^ (line 0228–0312). See Fig. 1 for location.

**Figure 3:**
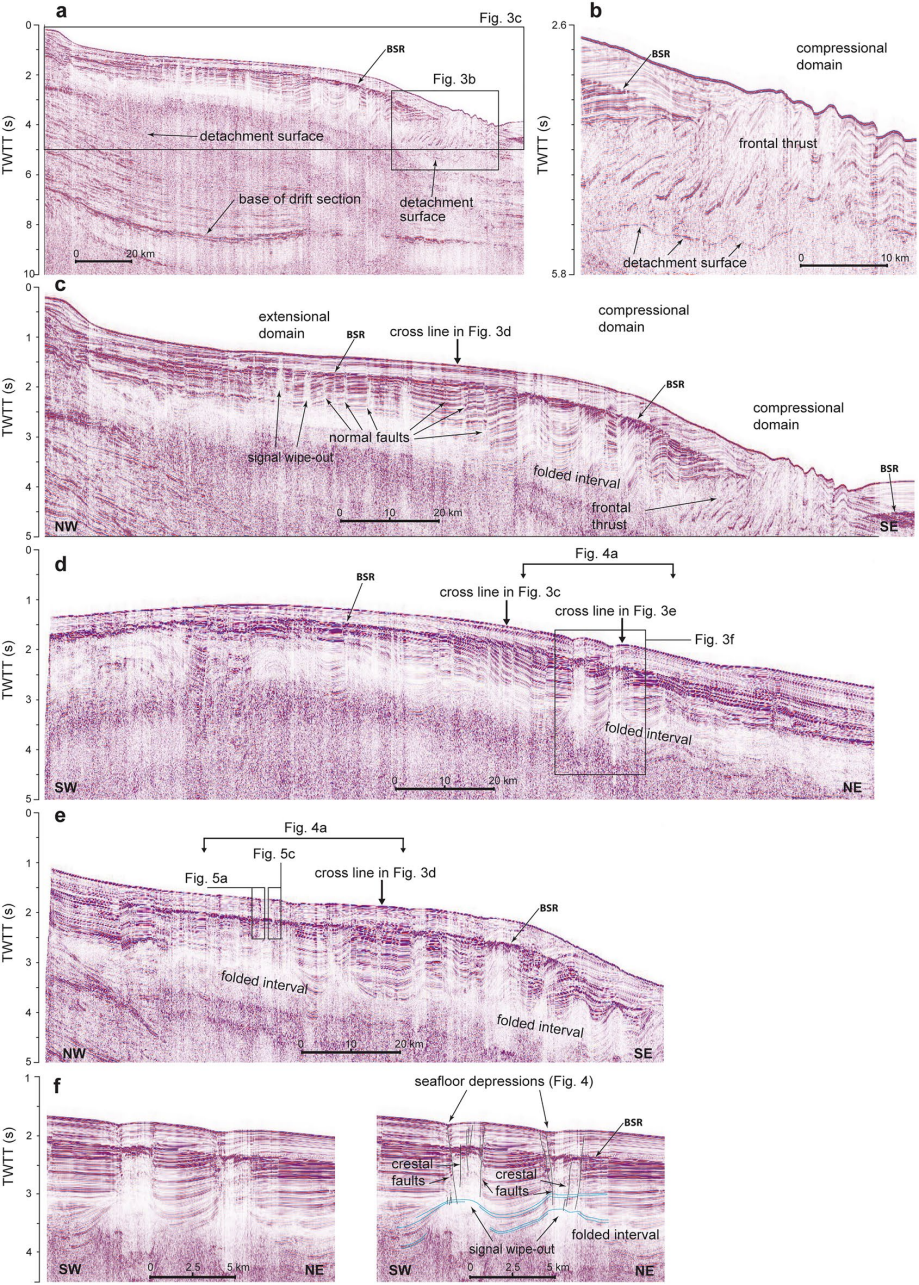
Seismic profiles illustrating tectonic and fluid migration structures within the Rio Grande Cone (see Fig. 1a for locations): (**a**) a deep profile imaging the main detachment surface of the Rio Grande Cone (RGC), the base of the drift section of the South Atlantic (Albian/Cenomanian^39^) and the bottom simulating reflector (BSR). (**b**) Detail of (**a**) showing the frontal thrusts and the detachment surface. (**c**) Detail of (**a**) showing the BSR and the extensional (upslope) and the compressional (downslope) structural domains, and associated structures (normal faults and the thrusts is a fold-and-thrust belt, respectively). Some normal faults are associated with signal wipe-outs possibly related to the presence of free gas; most terminate at the BSR and do not reach the seafloor. The frontal thrusts in the deepest part of the compressional domain form elevations of up to 200 m in seafloor relief over the Oligocene–Miocene overpressured strata. (**d**) Section showing structures rooted at the Miocene folded interval and reaching the seafloor (details in Fig. 2f). (**e**) Section showing another example of structures rooted at the folded Miocene interval and reaching the seafloor (details in Fig. 4a,c). (**f**) Detail of (**d**) showing uninterpreted section (left) and crestal faults (black lines, right) rooted on the anticlines in folded interval (right); note a seafloor depression associated with the crestal faults and the signal wipe-out zone connecting the buried anticlines to the seafloor. Light-blue lines are drawn to facilitate the correlation of seismic reflections in folded strata. See Fig. 1 for location of sections.

The Rio Grande Cone (RGC) consists of a 5 km-thick sediment pile that is thicker than adjacent areas^30^, i.e., a depocenter, formed by the accumulation of early Miocene-present fine-grained sediments in the Pelotas Basin, southern Brazil^31^ (Figs. 1 and 2). This depocenter, with seafloor relief above adjacent areas, hosts a large gas hydrate system identified on the basis of a near-continuous, widespread bottom simulating reflector (BSR) that extends over the limits of the RGC (at least 45,000 km^2^) at water depths ranging from 500 to 3500 m, but mostly at 200–300 m below seafloor (Fig. 3a)^32,33^. Previous work has shown that such BSRs mark the base of the GHSZ and the boundary between pore space with gas hydrate above and free gas below^34–36^. Simple calculations taking into account the sediment volume between the seafloor and the BSR, and a methane hydrate saturation of 1.5% suggest the existence of 22 trillion m^3^ or 780 tcf of methane in the area (i.e., ca. 12 GtC)^32^.

Rapid deposition since the Neogene has resulted in the gravitational collapse of the RGC above a detachment surface^41^ and its separation into two main structural domains (Fig. 2). There exists: (1) an extensional, upslope domain (dominantly < 1300 m water depth) with a series of proximal, slope-parallel listric normal faults; and (2) a compressional, downslope domain (dominantly > 2500 m water depth) with thrust faults and folding forming a slope-parallel belt^41^. Both the normal and thrust faults connect at depth to detachment surfaces developed in overpressured shale intervals of Oligocene–Miocene age at a depth of 2–3 km below the seafloor^39,40^.

**Figure 4:**
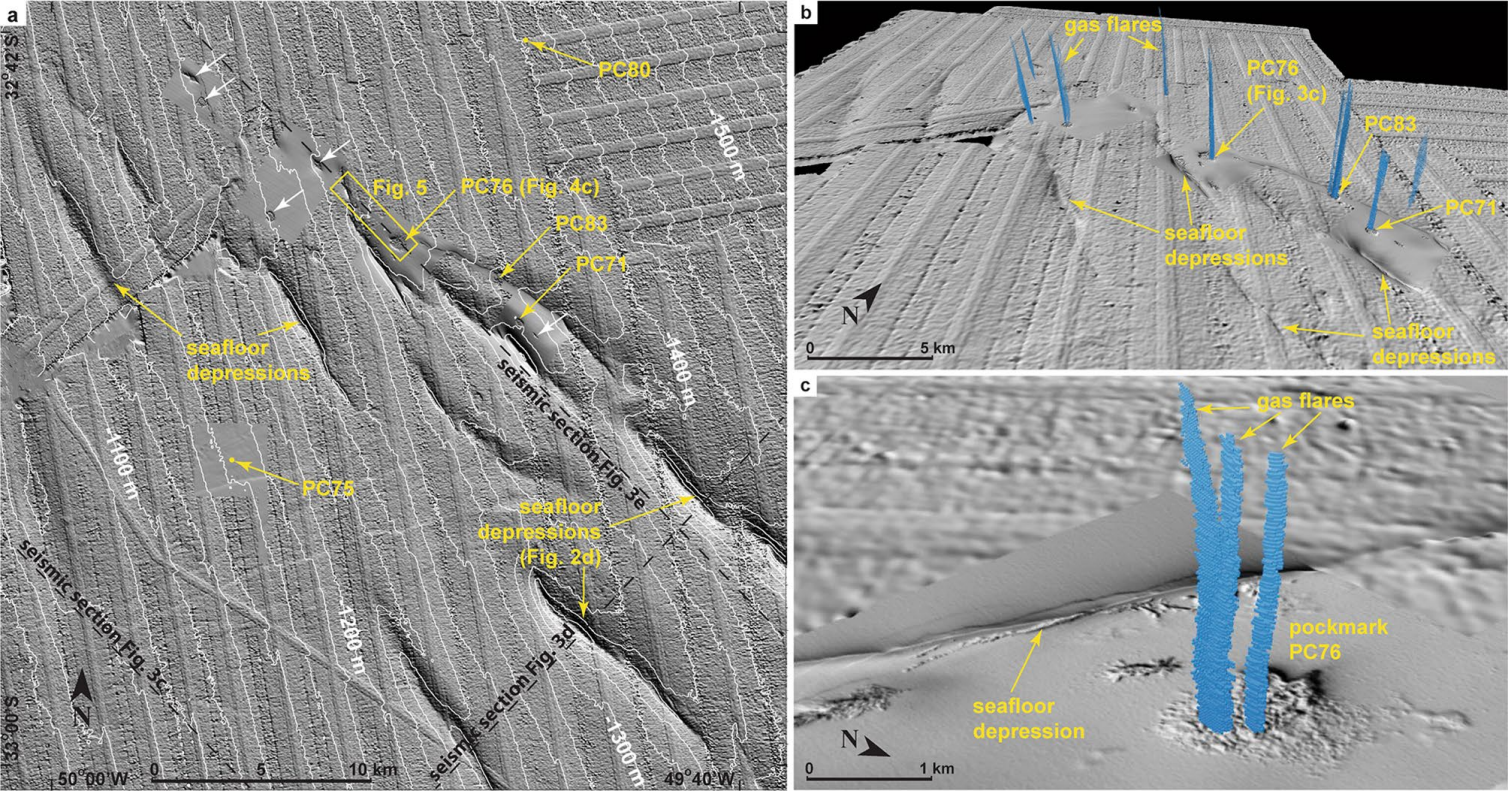
(**a**) Bathymetric map (shaded relief with isolines every 20 m) of part of the study area (see Fig. 1) obtained from a previous study^38^ showing aligned and elongate seafloor depressions, the trace of the used seismic profiles used in this study, and the locations of 5 piston cores obtained both in pockmarks (PC71, PC76 and PC83) and in background areas (PC75 and PC80). These depressions are the seafloor expression of crestal faults (see Fig. 2f). White arrows point to additional pockmarks in the area. A conductivity, temperature, depth profile of the water column was obtained in the same location of PC83. (**b**) 3D view of the study area showing sonar-detected water column gas flares (in blue) in relation to the seafloor depressions and pockmarks. Note the alignment of the gas flares along the NE–SW trend of main seafloor depressions. Flares in pockmarks near PC71, PC76, and PC83 were inspected with a remotely operated vehicle. Gas hydrate was recovered in all piston cores within pockmarks. (**c**) Detail of (**b**) showing multiple gas flares in pockmark PC76 and an adjacent seafloor depression. The bathymetry data was processed in Surfer (version 15; https://www.goldensoftware.com/products/surfer) and the assembly of bathymetry and gas flares was completed using QPS Fledermaus (version 7; https://www.qps.nl/fledermaus/).

The extensional and compressional domains of RGC show key features on seismic profiles (Fig. 3a–c). In places, strata adjacent to normal faults in the extensional domain show acoustic "wipe-outs" or a noticeable decrease in the seismic reflection amplitude. This is interpreted to represent zones of fluid migration and potential free gas^19,42,43^. Most of the wipe-outs, however, terminate below the BSR and do not extend to the seafloor (Fig. 3c). The compressional domain includes a distinctive subsurface folded interval of Oligocene–Miocene age^39^. The folded interval evolves downslope to imbricate thrusts that outcrop at the toe of RGC and form seafloor elongated hills with up to 200 m of relief (Fig. 3a–c). Buried anticlines at the top of the folded interval in places exhibit crestal normal faults that extend upwards to the seafloor (Fig. 3f). At the seafloor, these faults form NW–SE oriented depressions up to 40 m deep and occur together with a pockmark field interpreted as being formed by active gas venting in the area^38^ (Fig. 4).

**Figure 5:**
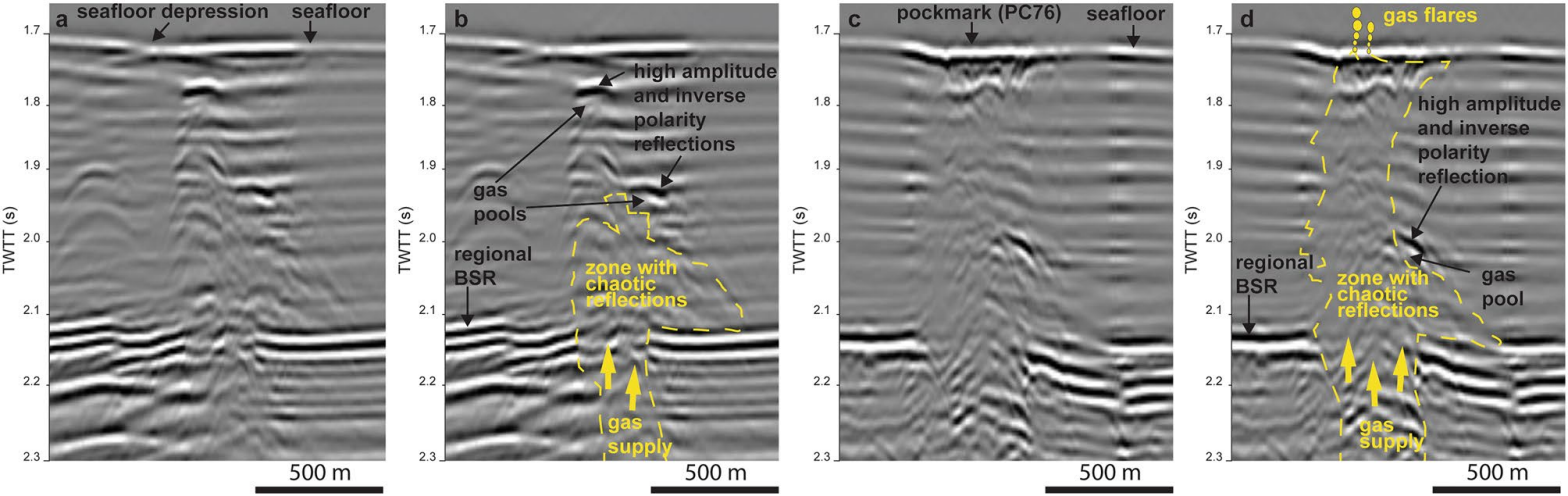
Uninterpreted (**a**, **c**) and respective interpreted (**b**, **d**) sectors of seismic profile in Fig. 3e showing a zone of low amplitude and chaotic reflections between the regional bottom simulating reflector (BSR) and the seafloor. Note the presence of reflections of high amplitude and reverse polarity reflections (in relation to the seafloor), which indicate the top of possible gas pools within the GHSZ (i.e., methane trapped as free gas in the hydrate structure and/or coexisting with gas hydrate and “dry” sediments, or both). Free gas is supplied from depth to the GHSZ across the amplitude loss interval or gap in the regional BSR. Gas flares were detected in the pockmark showed in (**c**) and (**d**), which has also been cored to recover gas hydrate^38^ (PC76; see Fig. 4a for location).

Beneath the pockmarks, crestal faults abruptly interrupt the regional BSR with a 500–700 m wide interval of seismic amplitude loss (Fig. 5). Above and below these intervals, a chaotic zone of mainly low seismic amplitude reflections rises from the base of the crestal faults (axes and tops of anticlines) towards the seafloor (Figs. 2f and 5), pointing out to stratal disruption and signal wipe-out due to rising gas-bearing fluids. In some places, high amplitude reflections with negative polarity can be recognised in the chaotic zone above the regional BSR (Fig. 5). We interpret these features as gas pockets within the regional GHSZ where methane is trapped as free gas in the hydrate structure^44^ and/or coexists with gas hydrate and “dry” sediments^45^, or both (Fig. 5).

The migration and accumulation of free gas along conduits across the regional GHSZ has been the focus of multiple studies^18,19,46,47^, with several mechanisms proposed to explain these phenomena. Rising of warm or high salinity fluids can locally shoal the GHSZ^43^, but the BSR on the RGC is abruptly interrupted instead of showing an upward deflection (a so-called "pluming BSR") as commonly observed in areas where fluids of different characteristics migrate upward along faults^35^. In areas of upward fluid migration, high rates of gas hydrate formation can generate extremely saline pore waters, which can lead to the development of chimneys that rise from the base of the GHSZ to seafloor with three co-existing phases (water, hydrate, and gas)^18^. However, such models imply salinities nearly twice standard seawater concentration^18^, which are not fully supported by available resistivity profiles in cores^48^ and porewater chloride concentration data in cores from the three studied pockmarks (Supplementary Data 1). In addition, high salinity pore waters within the GHSZ are not consistent with controlled source electromagnetic (CSEM) data available for the RGC, which shows high resistivity values within the seismically chaotic zones beneath pockmarks. Such high resistivity values are suggestive of high saturation of gas and/or gas hydrates (up to 60%)^48^. Although our data cannot exclude the three-phase system hypothesis, the high resistivity values and the not sufficiently high chloride concentrations found in the study area do not fully support the existence of high-salinity pore water and suggest that free gas in the GHSZ may be due to another mechanism, possibly linked to gas supply in excess of hydrate formation^20^. The gas is supplied via the mapped structures showing chaotic reflections underneath pockmarks; additionally, gas can be supplied by conducts that cannot be imaged on seismic^49^.

The CSEM data further supports the idea that systems allowing for gas migration through the GHSZ are ephemeral, because some of the observed pockmarks are not currently associated with high resistivity anomalies (indicating high gas or gas hydrate saturations) at depth^48^. These areas are interpreted as ancient emission loci that are no longer active^48^. In addition, high gas and gas hydrate saturations are observed in areas with no clear connection to seafloor seep structures^48^, and are therefore interpreted as systems yet to be fully developed, i.e., no full bypass of gas occurs through the GHSZ and conduits for gas are not imaged on seismic^49^. Present-day, fully developed systems in the RGC have high gas saturations underneath pockmarks^48^ and are commonly accompanied by flares in the water column. These observations suggest that seafloor methane emissions linked tectonic structures are episodic, and may be controlled, for instance, by the activity of the associated structures as observed in SE Japan^49,50^. It has been demonstrated that the regional tectonic regime (e.g., tension vs. strike-slip) controls the permeability and gas supply to faults and the related activity of seafloor methane seeps^51,52^. In large sediment-laden continental slopes, the activity of structures related to gravitational collapse is controlled by several factors^53,54^, including the response of the system to sediment loading and propagation of the deformation in the sedimentary package^55^, which, in turn, may control the activity of seafloor seeps.

## Seafloor emissions

Gas flares were identified in the water column over pockmarks using multibeam sonar data acquired over an area of 320 km^2^ (Fig. 4). Seafloor morpho-bathymetric imagery shows the pockmarks to be spatially associated with the NW–SE seafloor depressions, which are, in turn, connected in depth with gas wipe-outs and the crestal faults on top of the anticline structures of the folded Miocene interval (Fig. 2f). The sonar data reveals the presence of 12 giant gas flares rising to 900 m into the water column from eight pockmarks; up to three plumes can be seen to originate from a single pockmark (Fig. 4c). The sonar data also show that methane bubbles rise to water depths of ca. 610–650 m, which is the approximate upper limit of the methane hydrate stability zone calculated for the area (ca. 635 m, see “Methods”). Methane bubbles quickly dissolve at shallower water depths, presumably because a protective gas hydrate coating around the bubbles dissociates^3^. After dissolution, methane is likely to be consumed via aerobic oxidation in the water column^56^ and, therefore, may not reach the atmosphere at present. Such emissions, together with diffusion of gas through sediments, can potentially contribute to long-term ocean acidification and de-oxygenation^5,57^.

**Figure 6:**
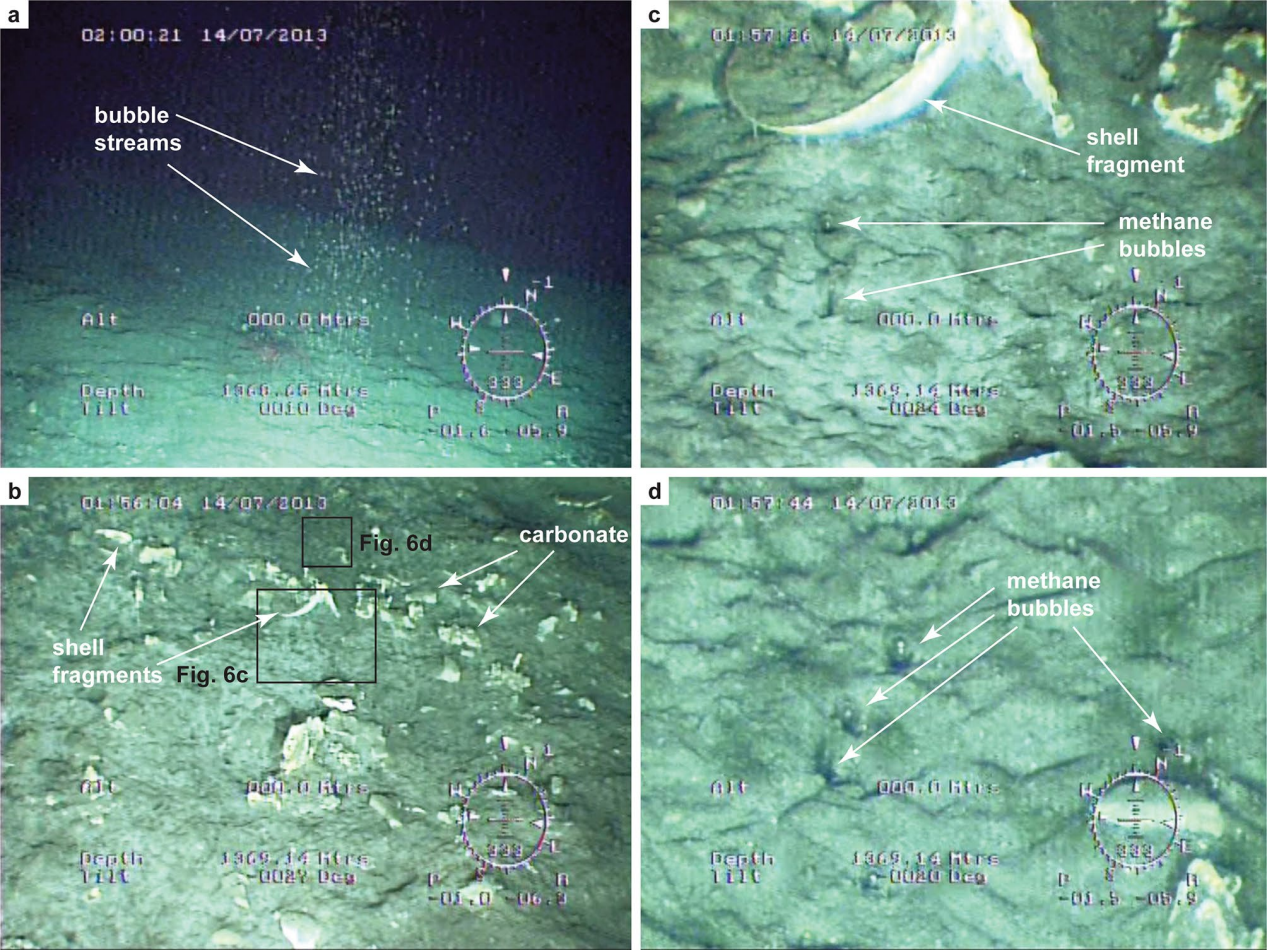
Seafloor photographs taken from a remotely operated vehicle (ROV) showing seafloor methane emission sites: (**a**) gas flare formed by a cluster of bubble streams (diameter of the cluster is ca. 1.5 m), (**b**) detail of the seafloor nearby flares with fragments of shells and carbonate concretions, (**c**) and (**d**) methane bubbles of ca. 0.5 cm in diameter venting from the seafloor (bubbling rate varies considerably among streams, from 0.5 to 7 bubbles per second or ca. 0.03–0.5 cm^3^ s^−1^ of methane in each stream). The shell fragment identified in (**b**) and (**c**) is ca. 10 cm in diameter. The photos were taken in the gas flare in the pockmark near the PC83 piston core (see Fig. 4a for location and Supplementary Videos 1 and 2 for a more complete documentation of the flares).

Gas flares were visually observed at the seafloor in three pockmarks (PC71, PC76, and PC83; see Fig. 4 for location) using a remotely operated vehicle (ROV; Fig. 6; Supplementary Movies 1 and 2). Each flare identified on sonar data corresponds to 5–32 individual bubble streams. A gas hydrate coating was observed to form on each bubble at ca. 20 cm above seafloor, and carbonate concretions and shell fragments were found on the seafloor around the streams (Fig. 6). The bubbling rate varies considerably among streams, from less than one to seven bubbles per second. The lowest methane emission rate was observed in a flare comprising five bubble streams (3.88 Mg year^−1^) spread over an area of 1 m^2^ and the highest in a flare with 32 bubble streams (25.83 Mg year^−1^) in an area of 6 m^2^ (see Supplementary Note 1). These values are similar to the emission rates calculated for individual flares in the central province of the Nile deep-sea fan (3.69–36.9 Mg year^−1^)^58^. Extrapolation of bubbling rates measured at the three selected flare sites to all 12 flares identified in the study area results in a total methane emission rate in gas phase between 47 and 310 Mg year^−1^. This is twice the total methane emission rate found for all of the 394 flares at the feather edge of the GHSZ on the upper slope of the RGC, ca. 90 km to the south-west (Fig. 1)^37^, but nearly half of the average rate found for 452 flares on the continental margin west of Svalbard (848 Mg year^−1^)^59,60^. It is, however, three times the total estimated methane emission rate for the entire northern US Atlantic margin (15–90 Mg year^−1^) where ca. 570 flares have been mapped between Cape Hatteras to Georges Bank)^2^ through an area of 94,000 km^2^. The presence of carbonate concretions in the seep sites of the RGC further indicates that methane emissions in the area started, at least, thousands of years ago^61^.

## Origin of methane

**Figure 7:**
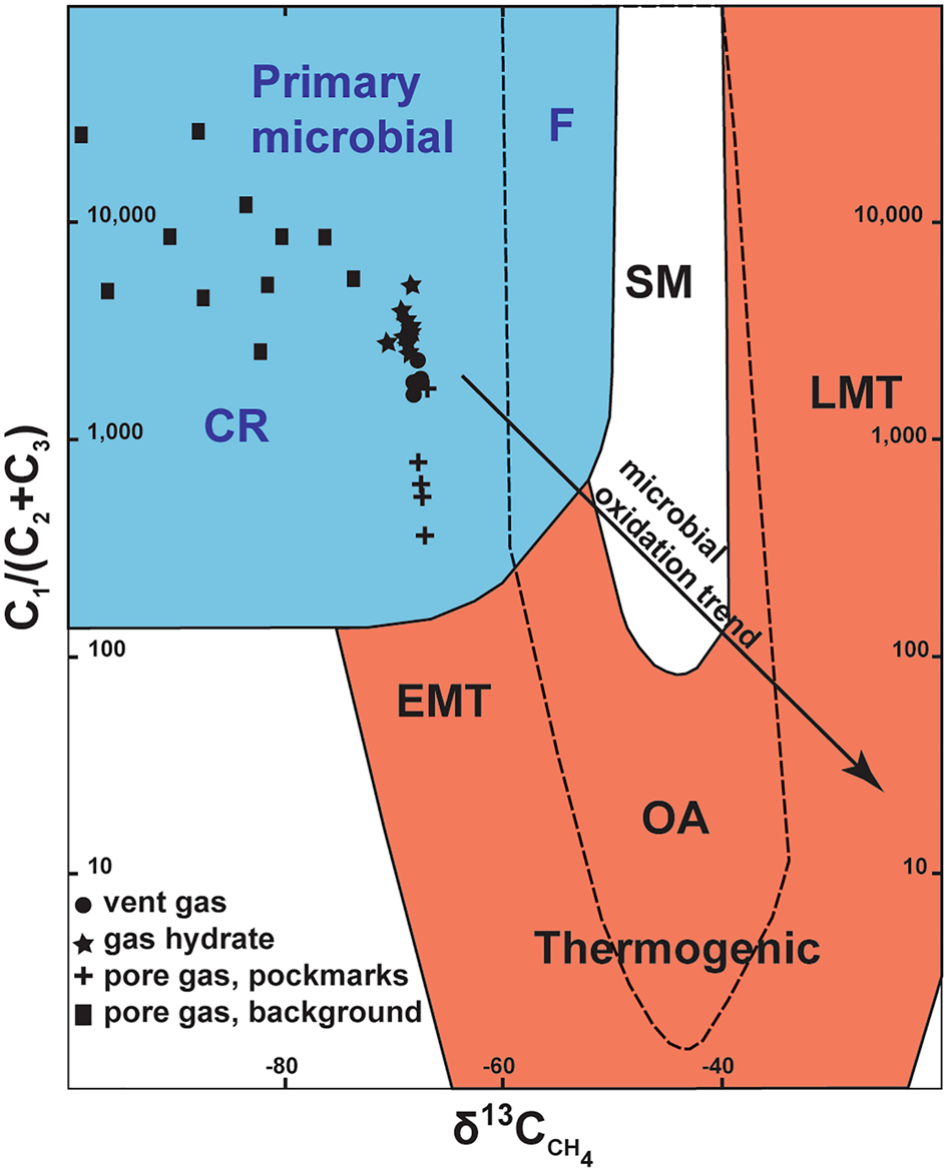
Diagram showing the relationship between carbon stable isotope composition (δ^13^C) of methane and the methane/ethane + propane ratio (C1/(C2 + C3)) of different gas samples analysed in this study. All samples indicate a primary microbial origin for methane. Gas hydrate and vent gas samples show a deviation from pore gas samples in pockmarks possibly owing to gas hydrate precipitation and anaerobic oxidation of methane, while background samples show a trend typical of microbial oxidation. Detectable quantities of the ^14^C isotope were found in gas samples in sediment pores, but not in gas hydrates and vent gas. The genetic fields (CR = CO_2_ reduction, F = methyl-type fermentation, SM = secondary microbial, EMT = early mature thermogenic gas, OA = oil-associated thermogenic gas, and LMT = late mature thermogenic gas) are from a global compilation of revised genetic diagrams for natural gases^65^.

Chemical and isotopic analyses of gases in gas bubbles collected during ROV surveys, and in sediment pores and in hydrates recovered in five piston cores in the area (see Fig. 3 for location), reveal the dominant hydrocarbon (> 99%) to comprise methane of microbial origin (Fig. 7; Supplementary Data 2). Gas bubbles have a fairly homogeneous stable isotopic composition (δ^13^C from − 68.2 to − 66.54‰) and no detectable ^14^C isotope, suggesting a homogeneous source below the seafloor and little modification during migration. Although we cannot exclude some gas contribution from strata below, these observations concur with seismic evidence of signal wipe-outs consistent with the rise of gas-bearing fluids from folded and overpressured Oligocene–Miocene interval (2–3 km depth) located at the roots of the crestal faults (Fig. 2f). Gas migrating from depth via fault systems is also the probable source for gas hydrate deposits indicated by low resistivities within the seismically chaotic columns or dim zones observed beneath pockmarks. Methane trapped in gas hydrates has a similar, but slightly more ^13^C-depleted carbon stable isotopic composition (δ^13^C from − 70.27 to − 67.84‰) when compared to the methane bubbles released in flares (Fig. 7), with neither having a detectable ^14^C isotope. Heavier hydrocarbons such as ethane and propane occur in very low concentrations (< 0.016 mM; Supplementary Data 2) and, under such conditions, occur as dissolved phases that are not a significant part of gas hydrates^62^. In contrast, gas mixtures in pores of sediment sampled inside pockmarks show a slight enrichment in heavier hydrocarbons relative to the gas bubbles in flares (Fig. 7). This is attributed to the uptake of methane into gas hydrate structures, and the preferential consumption of methane at shallow depths below the seafloor via anaerobic oxidation of methane^63^. The similar range of δ^13^C values (δ^13^C from − 68.46 to − 66.66‰), together with the presence of the ^14^C isotope, indicates that the gas mixture in sediment pores within pockmarks contain methane produced during recent (< 43,500 years B.P.) methanogenesis in addition to older methane flowing from depth. A greater range of δ^13^C values and stronger depletion of the stable ^13^C isotope (δ^13^C ranging from − 98.37 to − 73.3‰), and a higher concentration of the ^14^C isotope occur away from the pockmarks (Fig. 7), indicating a stronger contribution of methane produced locally during recent methanogenesis in sediment pores. This pattern has also been observed adjacent to hydrate-rich areas with structures focusing fluid flow in the Japan Sea^64^. In addition, the chemical and isotopic compositions of methane in sediment pores in these areas show a potential microbial oxidation trend (Fig. 7), as expected for gas mixtures at shallow depths below the seafloor^65^.

## Loci for seafloor methane emissions

Faults on top of thrust anticlines in the compressional zone are the main conduits for gas migration to the seafloor in the RGC. These faults control the spatial distribution and activity of methane leakage structures such as pockmarks and associated gas flares. However, no available data indicate active gas seeps near the anticlines at the toe of RGC, thought thrust-related structures are common sites for gas leakage in other gravity complexes. Seismic and multibeam sonar data from the Amazon deep-sea fan, for instance, show that thrust-related anticlines and associated strike-slip faults rising to the seafloor control the distribution of 17 gas flares over an area of 18.000 km^2^ in the compressional domain of the upper fan (water depths between 1000 and 1800 m)^11^. A similar pattern of seafloor structures can be seen on seismic profiles from the lower Niger deep-sea fan (water depth ca. 2500 m)^66^, where abundant seeps occur mostly in the hanging-wall of anticlines within the compressional domain of the fan. There, one major seep was recognised at the upper tip of a fault connecting the fore-thrust with the seafloor^66^. Interestingly, seafloor methane leakage sites (mostly mud volcanos) have also been recognised in the middle to upper parts of the Niger fan linked to deep, listric normal faults in the extensional domain (water depths < 1000 m)^67^. There is no clear indication of seafloor seeps in the extensional domain of the RGC, except for the system associated with hydrate dissociation at the feather edge of the GHSZ^37,38^. The geometry of listric faults favours dilation and therefore focusing of fluid flow and fluid scape at their upper tips^68^ in the extensional domain of the RGC, which corroborates with the signal wipe-outs observed in the seismic data (Fig. 2a). However, most of the gas is retained at the base of the GHSZ and does not reach the seafloor (Fig. 2a) suggesting: (1) transport dominated by diffusion, (2) an insufficient gas flow to cross the GHSZ^20^ and/or (3) a period of fault inactivity and diminished fluid flow^55^.

**Figure 8:**
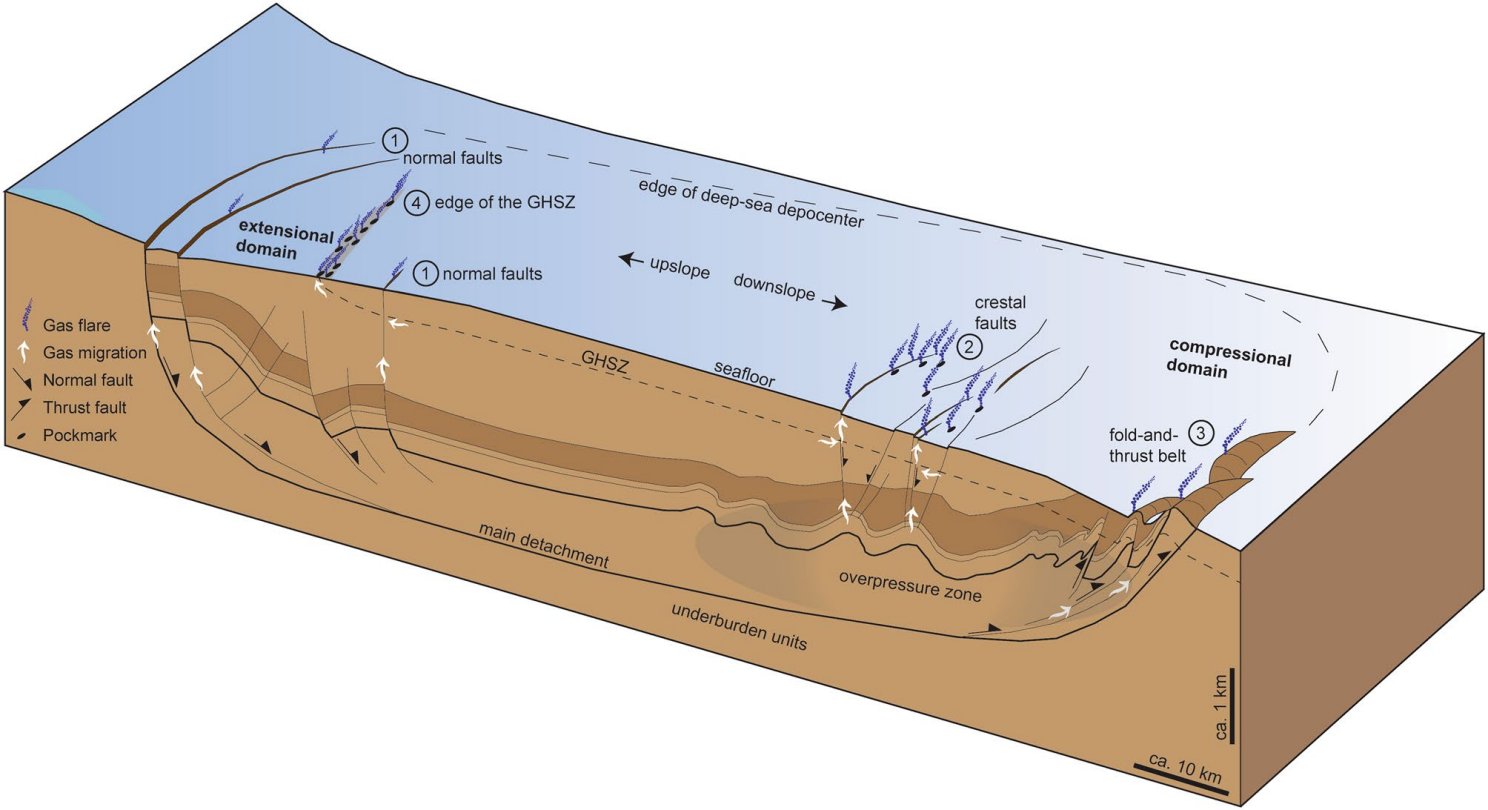
Schematic diagram summarising the probable loci of seafloor methane release associated with the gravitational collapse of sediment-laden continental slopes. Methane leakage occurs in both the extensional and compressional domains, preferentially at the intersection of the following structures with the seafloor: (1) listric normal faults in the extensional domain, (2) crestal faults on top of folds and (3) thrust and related strike-slip faults in the compressional domain. Another potential locus for seafloor methane emission, not related to gravitational collapse, is (4) the feather edge of the gas hydrate stability zone (GHSZ). The figure was elaborated in Adobe Illustrator (version 26.0.1; https://www.adobe.com/illustrator).

Our study of the RGC, supplemented by evidence from the Amazon and Niger fans, allows us to elaborate a novel, predictive model for the distribution of potential seafloor methane emission sites in other gravitational deep-sea complexes in passive margins (Fig. 8). Our model may also help to understand the distribution of seafloor seeps in other tectonic settings^49,50^. In the RGC, significant quantities of methane (3.88–25.83 Mg year^−1^) leak from those structures from a relatively small seafloor area (320 km^2^). Higher emission rates may be encountered in larger deep-sea accumulations such the Amazon and Niger fans, or in dozens of other depocenters around the world^28^ affected by gravitational collapse. A systematic review of geophysical data from such areas may quickly reveal the existence of many yet unknown sites of massive seafloor methane leakage.

## Methods

### Acoustic surveys

Slope-crossing (dip) and slope-parallel (strike), 2D multichannel seismic profiles (dominant frequency 40 Hz) loaded in IHS Kingdom Suite software were used to identify and map regional tectonic structures and the bottom simulating reflector (BSR). Ship-borne multi-beam echo sounder (MBES) installed onboard research vessels Marechal Rondon (model Reson Seabat 7150), and Marion Dufresne (model Thomson Seafalcon 11) were used to obtain high-resolution bathymetric data of the area. Water column acoustic imagery was acquired in January 2014 using the MBES installed on the research vessel Rig Supporter (Simrad EM 1002). These data was used to map gas flares associated with fluid escape structures on the seafloor. Sonar data was processed, integrated and visualised with IVS-3D Fledermaus software with water column module.

### Flare inspection, seep rate and gas sampling

Three previously identified gas flares showing strong acoustic backscatter signal were selected for visual inspection and gas sampling using a remotely operated vehicle (ROV; Mohican Inspection Class, equipped with high-resolution camera and two manipulators) deployed from the research vessel Marion Dufresne in July 2013. A high precision acoustic positioning system (HiPAP) was used to establish the ROV’s position during the dives, and the exact location of the gas flares on the seafloor were determined using the ROV’s front sonar. Gas bubbles were sampled with a deep-water fluid sampler device consisting of a transparent (acrylic) funnel with markings to estimate bubble size and gas volume, and a 300 mL stainless-steel bottle with vacuum. The funnel and the bottle were connected to a ball-valve operated by the ROV’s manipulators. The device was positioned on top of the bubbling sites; when the funnel was full, the valve was opened and then closed, allowing the gas bubbles to flow and to be trapped into the bottle. Gas seepage was quantified by inspecting the video records and counting the number of bubbles venting out from the seafloor per unit of time^37^. The calculations assume no temporal variability of advective flux on timescales longer than the observed time (ca. 1 h). Measured bubble diameter was 0.5 cm and the quantity of methane *n* (moles) in each bubble was calculated based on the universal gas law (n = PV/ZRT), where *P* is pressure (13.5 MPa), *V* is the bubble volume (0.065 cm^3^), *Z* is the compressibility of methane (0.7426) calculated by the Peng − Robinson equation of state^69^, *R* is the gas constant, and *T* is the bottom water temperature (3.25 °C). The mass of methane in each bubble was calculated based on the number of moles (obtained from the universal gas law) and the molar weight of methane. The emissions were then estimated based on the number of bubbles per unit of time. The bottom water temperature and pressure were obtained from a conductivity, temperature, depth (CTD Sea-Bird Electronics SBE 911plus) profile acquired in the area near PC83 (see Supplementary Data 3 and Fig. 4a for location). The CTD (temperature) data was also used to calculate the depth of the upper limit of the methane hydrate stability zone in the water column using the equilibrium equation of pure methane in seawater^70^.

### Chemical and isotopic composition of gas

Sediment samples (ca. 200 cm^3^) were collected every 1.5 m in all piston cores for gas analyses. Samples were placed in gas-tight containers (Isojars), together with 200 cm^3^ of distilled water and 10 drops of Zephiran Chloride bactericide. The remaining 200 cm^3^ in the jar was left for the formation of headspace. Samples of gas hydrates were also added into jars. The chemical composition of headspace and gas bubbles collected in flares were determined using a Shimadzu gas chromatograph (GC) model GC-2014 equipped with a capillary column VP-Plot Alumina/KCl, 30 m × 0.53 mm, and flame ionisation detector. Helium was used as carrier gas (flow rate of 5 mL min^−1^) and the oven temperature was set to 200 °C. Carbon stable isotopic composition was determined with a GC (Thermo Scientific) coupled to an isotopic ratio mass spectrometer (Thermo Scientific DELTA-V Plus) via a Thermo GC IsoLink and Conflo IV interfaces. The GC has a 30 m × 0.32 mm fused silica column, Carboxen Plot 1006, and a heating ramp of 70–150 °C was set over 30 min. The isotopic ratio is reported using the delta notation (δ^13^C) relative to the international Vienna Pee Dee Belemnite standard (V-PDB). The radiogenic ^14^C isotope analyses of methane were performed in the venting gas bubbles, gas in hydrates and gas in sediment pores using an accelerator mass spectrometry in the Beta Analytic laboratory in Florida, U.S.A. The modern reference standard was 95% the ^14^C activity of the National Institute of Standards and Technology (NIST) Oxalic Acid (SRM 4990C) and calculated using the Libby carbon half-life (5568 years).

## Supplementary Information

Supplementary Legends.

Supplementary Information.

Supplementary Video 1.

Supplementary Video 2.

Supplementary Information 1.

Supplementary Information 2.

Supplementary Information 3.

## Data Availability

All relevant data are included in the Supplementary material to this article.
