# Supplementary material for: Gravity complexes as a focus of seafloor fluid seepage: the Rio Grande Cone, SE Brazil
Source: Sci Rep. 2023 Mar 21;13:4590. doi: 10.1038/s41598-023-31815-1 (PMC10030975; doi:10.1038/s41598-023-31815-1)
Supplement: Supplementary file 1 — Supplementary Legends. [file 41598_2023_31815_MOESM1_ESM.pdf]

Titles and captions for supplementary files for GRAVITY COMPLEXES AS A FOCUS OF SEAFLOOR FLUID SEEPAGE: THE RIO GRANDE CONE, SE BRAZIL by Ketzer et al.

Supplementary data 1 – Chloride concentration profiles.
Chloride concentration (pore water) vs. depth for three piston cores obtained in the studied pockmarks of the Rio Grande Cone (PC71, PC76, PC83). Note that the concentrations vary between 11,643-27,000 ppm (up to ca. 40% higher than standard seawater). Chloride concentration was obtained via ionic chromatography (Thermo Scientific, Dionex ICS-5000 dual pump system) after dilution with deionized water.

Supplementary data 2 – Chemical and isotopic composition of gases.

Methane and ethane + propane concentrations, and carbon isotopic values of methane for vent bubbles, gas hydrate and pore gas samples (see Figure 3 for location).

Supplementary data 3 – Temperature vs. depth profile of the water column.

Temperature vs. depth profile of the water column obtained in pockmark PC83 (see Figure 3 for location) with a conductivity, temperature, depth (CTD) cast.

Supplementary video 1 – Gas flare.

Video showing gas flare with several bubble streams in pockmark PC83 (see Figure 3 for location). Note hydrate coating on bubbles as they rise in the water column (26-36 seconds).

Supplementary video 2 – Details of bubble streams in gas flare.

Video showing details of bubble streams in a gas flare in pockmark PC83 (see Figure 3 for location).
