# Supplementary material for: Gravity complexes as a focus of seafloor fluid seepage: the Rio Grande Cone, SE Brazil
Source: Sci Rep. 2023 Mar 21;13:4590. doi: 10.1038/s41598-023-31815-1 (PMC10030975; doi:10.1038/s41598-023-31815-1)
Supplement: Supplementary file 2 — Supplementary Information. [file 41598_2023_31815_MOESM2_ESM.pdf]

Supplementary information for GRAVITY COMPLEXES AS A FOCUS OF SEAFLOOR FLUID SEEPAGE: THE RIO GRANDE CONE, SE BRAZIL by Ketzer et al.

Supplementary Note 1

Information about inspected gas flares with a remotely operate vehicle. Number of bubble streams per flare and bubbling rates were used to calculate methane advective flux assuming bubble diameter of 0.5 cm. The quantity of methane n (moles) in each bubble was calculated based on the universal gas law and converted to mass (see Methods).

Flare 1 – Area 1 m^2

Stream no.	number of bubbles per second	emissions $cm^3\ h^{-1}$	emissions $Mg\ yr^{-1}$
1	2	471.238898	0.5244
2	3	706.858347	0.7866
3	2.5	589.048623	0.6555
4	3	706.858347	0.7866
5	4.3	1013.16363	1.1275
Total	14.8	3487.16785	3.8806

Flux = 2.4×10^4 mmol $cm^{-2}\ yr^{-1}$

Flare 2 – Area 6 m^2

Stream no.	number of bubbles per second	emissions $cm^3\ h^{-1}$	emissions $Mg\ yr^{-1}$
1	4	942.477796	1.04881687
2	0.5	117.809725	0.13110211
3	1	235.619449	0.26220422
4	2	471.238898	0.52440844
5	4	942.477796	1.04881687
6	7	1649.33614	1.83542953
7	2	471.238898	0.52440844
8	7	1649.33614	1.83542953
9	4	942.477796	1.04881687
10	4	942.477796	1.04881687
11	2	471.238898	0.52440844
12	1	235.619449	0.26220422
13	4	942.477796	1.04881687
14	0.5	117.809725	0.13110211
15	0.5	117.809725	0.13110211
16	7	1649.33614	1.83542953
17	4	942.477796	1.04881687
18	2	706.858347	0.78661266
19	7	1649.33614	1.83542953
20	2	471.238898	0.52440844
21	4	942.477796	1.04881687
22	4	942.477796	1.04881687
23	4	942.477796	1.04881687
24	0.5	117.809725	0.13110211
25	1	235.619449	0.26220422
26	4	942.477796	1.04881687
27	4	942.477796	1.04881687
28	2	471.238898	0.52440844
29	4	942.477796	1.04881687
30	2	471.238898	0.52440844
31	2	471.238898	0.52440844
32	0.5	117.809725	0.13110211
Total	97.5	23208.5157	25.8271155

Flux = 2.7×10^4 mmol $cm^{-2}\ yr^{-1}$
